# 614. Sociodemographic and Clinical Factors Associated with Post-burn Mental Health Diagnosis and Treatment

**DOI:** 10.1093/jbcr/irag033.428

**Published:** 2026-04-06

**Authors:** Connie J Tian, Peggy J Ebner, Haig A Yenikomshian, Sarah A Stoycos

**Affiliations:** Keck School of Medicine, University of Southern California, Los Angeles, CA; Keck School of Medicine, University of Southern California, Los Angeles, CA; Division of Plastic and Reconstructive Surgery, Keck School of Medicine, University of Southern California, Los Angeles, CA; Keck School of Medicine, University of Southern California, Los Angeles, CA

## Abstract

**Introduction:**

Burn injuries are strongly associated with anxiety, traumatic stress, and depressive disorders, which can negatively impact recovery and long-term quality of life. However, predictors of post-burn mental health diagnosis (MHD) and treatment remain poorly characterized. This study aims to identify sociodemographic and clinical factors associated with new MHDs and subsequent treatment in a large national cohort.

**Methods:**

Adults with burn injuries over the past 20 years were identified in the TriNetX research network. Patients with prior diagnoses of anxiety, depression, adjustment, or post-traumatic stress disorder (PTSD) were excluded. The primary outcome was a new MHD diagnosis within 2 years of burn injury. The secondary outcome was pharmacotherapy or psychotherapy treatment among those newly diagnosed. Propensity matching adjusted for sociodemographic and clinical covariates, and odds ratios (OR) with 95% confidence intervals (CIs) were calculated.

**Results:**

Among 583 626 patients, female sex (OR 1.97), White race (OR 1.49), and English language (OR 1.87) were associated with higher odds of new MHDs. Hypertension (OR 2.20), substance use disorder (OR 2.06), and burn total body surface area (TBSA) greater than 30% (OR 1.5) also increased risk. Mechanical ventilation and ICU admission within 30 days of injury were associated with lower odds of depression and anxiety but higher odds of PTSD. Among 23 404 patients with new MHDs, 20.1% received pharmacotherapy and 5.0% received psychotherapy. White (OR 1.34) and non-Hispanic (OR 1.25) patients were more likely to receive pharmacotherapy. Hypertension (OR 1.32), cardiovascular disease (OR 1.43), and substance use disorder (OR 1.41) were also predictive of pharmacotherapy treatment. No significant disparities were observed in psychotherapy use.

**Conclusions:**

Both sociodemographic and clinical factors significantly influence mental health diagnosis and treatment after burn injury. These findings underscore the need for routine, culturally-informed screening that prioritizes high risk groups to ensure equitable care and timely treatment.

**Applicability of Research to Practice:**

This study highlights sociodemographic and clinical risk factors for post-burn mental health disorders. Implementing routine, culturally-informed screening may promote equitable care and improve overall outcomes for all burn patients.

**Funding for the study:**

This work was supported by NIDILRR (grant 90DPBU0007) and the National Institute of Mental Health (1K23MH141296-01). The contents do not necessarily represent the policy of NIMH, NIDILRR, ACL, or HHS, and endorsement by the Federal Government should not be assumed.

